# Removal of a Foley catheter misplaced into the ureter by percutaneous puncture: a rare case report

**DOI:** 10.1186/s12894-022-01057-w

**Published:** 2022-07-09

**Authors:** Peng-Fei Qin, Wan-Zhang Liu, Bin-Bin Yang, Kai-Ning Lu, Jun-Hai Qian, Jia-Sheng Hu, Yue Cheng

**Affiliations:** 1 School of Medicine, Ningbo University, #818, Fenghua Road, Ningbo, 315010 Zhejiang China; 2 Department of Urology, Ningbo First Hospital, #59, Liuting Street, Ningbo, 315010 Zhejiang China

**Keywords:** Aberrant catheterization, Percutaneous puncture, Complication, Mechanism

## Abstract

**Background:**

The incidence of aberrant catheterization into a ureter is extremely low, and there is a 20% chance that the balloon cannot be deflated. Regrettably, the mechanism underlying this complication remains unknown. There has been no reported case of a Foley catheter successfully removed from the ureter via percutaneous puncture.

**Case presentation:**

A 86-year-old man complained of increasing abdominal pain after an 18F Foley catheter was inserted into his urethra. His attending physician attempted but failed to deflate the balloon. A bedside ultrasound and CT scan revealed that the catheter tip was in the right lower ureter. Several measures, including cutting the catheter and inserting a rigid guidewire, were then attempted but failed to deflate the balloon. Finally, the inflated balloon was punctured with a PTC needle under ultrasound-guidance, and the misplaced Foley catheter was removed. Two days after the pelvic drainage tube was removed, the patient was discharged.

**Conclusion:**

This is the first reported case of a Foley catheter being removed from the ureter via percutaneous puncture. The mechanism by which the balloon is unable to deflate may be related to the passive twist of the catheter. In such a case, an overall assessment of the patient's condition should be performed, and non-invasive to invasive interventions should be phased in.

## Introduction

As a widely practiced clinical procedure, urethral catheterization is regarded as relatively safe. The occurrence of aberrant catheterization into a ureter is extremely rare, with only 26 cases reported worldwide [1–3]. Failure to remove the catheter was reported in five of these cases, which could lead to hydronephrosis, ureter rupture, and septicemia [4]. There is, however, no convincing explanation for why the aberrant catheter cannot be removed. Herein, we present a case in which a Foley catheter was misdirected into the right ureter and could not be removed. The catheter was eventually removed after a percutaneous puncture was performed. To our best knowledge, this is the first case of a catheter successfully removed from the ureter using this procedure.

### Case presentation

**Fig. 1:**
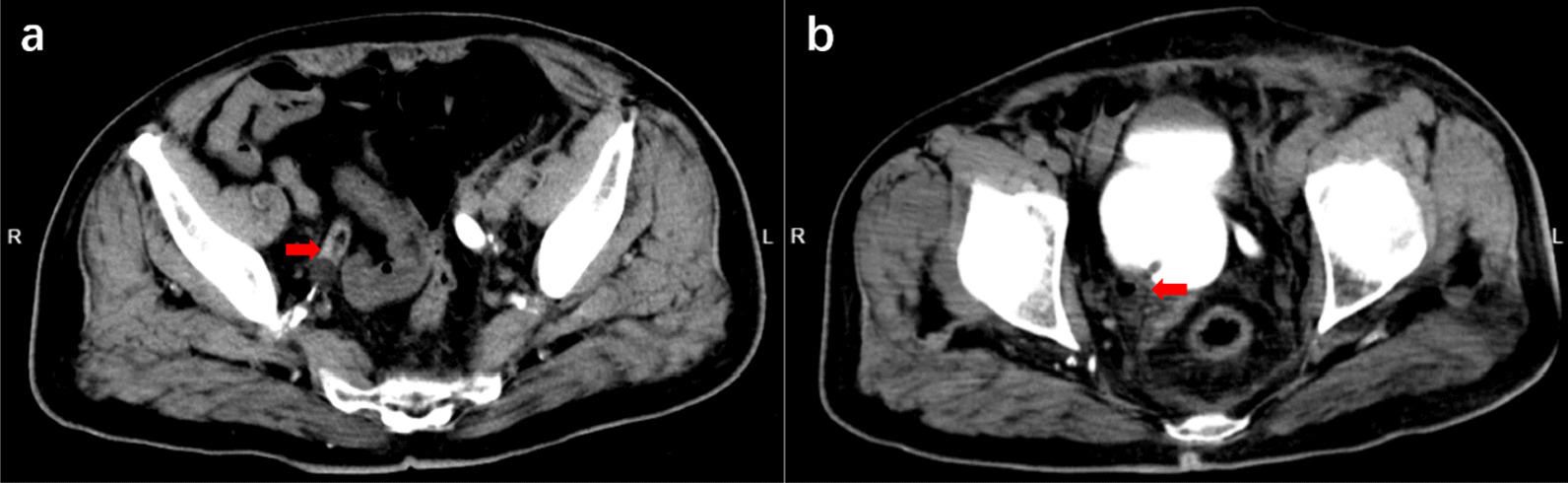
Images from computed tomography. **a** The tip of the Foley catheter is located in the right lower ureter. **b** The saline-filled balloon in the ureter cavity

A 86-year-old man was admitted to our hospital's cardiovascular surgery department with arteriosclerosis obliterans of the lower limbs. He had a history of bladder cancer and had a transurethral resection two years ago, which was followed by repeated intravesical instillation. Following the insertion of an 18F Foley catheter into his urethra by an intern, the patient complained of increasing abdominal pain. His attending physician then tried to remove the catheter, but the balloon could not be deflated. We were invited for a consultation and noticed that there was little urine flow from the catheter. In an attempt to deflate the balloon, the foley catheter was cut, but it failed. The catheter tip was found in the right lower ureter on bedside ultrasound and CT, along with bilateral hydroureter and hydronephrosis (Fig. 1). A Zebra guidewire was inserted into the balloon channel in an attempt to puncture the balloon, but still failed. Finally, a PTC needle (18G) was used to perform an ultrasound-guided percutaneous puncture through the puncture site in the right lower abdomen. The inflated balloon was visualized, and the needle was inserted into the right side of the bladder, slowly approaching the balloon. The misplaced catheter was removed after the balloon was punctured. A drainage tube was indwelled in the right pelvic cavity and a new Foley catheter was inserted. Over the next week, various conservative treatments including antibiotics and analgesics were administered. There was no significant hydronephrosis in either renal pelvis on the new CT scan, and no effusion in the abdomen or pelvis. During the 2-day observation period following the removal of the new catheter and drainage tube, the patient was discharged without significant urination abnormalities.

## Discussion and conclusion

Misdirection of an Indwelling Foley catheter into the ureter is a rare complication, and it appears to be linked to female gender, neurogenic bladder, and sensory disorder [5]. Despite the absence of these factors in this case, the bladder of this patient has an abnormal morphology. His history of bladder surgery and intravesical instillation could be the cause of anatomical changes in his bladder, such as an enlarged ureteral orifice, which may make it easier for a Foley catheter to be directed into the ureter. According to our data, about 20% of the balloons in the 26 cases of catheter misplacement into the ureter that have been reported so far have failed to deflate [1–3]. This suggests that a misplaced balloon may make deflation more difficult, and that the catheter's quality may not be to blame. We found a "twisting" shape of the catheter misplaced in the ureter based on CT and ultrasound images, similar to that reported by Cho et al. [3]. This "twist" may compress the balloon channel inside the catheter, making it impossible to deflate the balloon. This situation was simulated in vitro in our lab, and we found that when the distal catheter was twisted 180 degrees, the balloon channel was almost completely occluded. The balloon is relatively fixed as it inflates in the ureter cavity. The distal part of the catheter passes through the ureter orifice and neck of bladder, causing it to “twist” in the distal part. The inability to deflate the misplaced balloon may be explained in part by this "twist" mechanism. At the same time, the Foley catheter blocked the urethra, preventing urine from draining from the bladder. The high pressure was transmitted upwards, which explains the patient's hydronephrosis and hydroureter on the left side.

A table was created from five reported cases in which a catheter was misdirected into the ureter and the balloon could not be deflated (Table 1). Similarly, various measures were taken to deflate the balloon in these cases. The simplest method is to cut the catheter, assuming that the balloon will deflate passively once the valve is removed, but this is rarely successful. In a few cases, a non-invasive attempt to puncture the balloon by inserting a stiff guide wire into the balloon channel was successful [6]. Unfortunately, our guide wire coiled before reaching the balloon, probably due to a twisted distal catheter. Following the failure of previous attempts, endoscopic surgery and percutaneous puncture are considered to be effective solutions [3]. Both ureteroscopes and cystoscopes can provide a clear view of the position of the catheter in the body, and can be used to deflate the balloon with biopsy forceps, electrodes, and laser fibers [2, 7, 8]. We tried ultrasound-guided percutaneous puncture instead of ureteroscopy because it was midnight, there was no ureteroscope available, and the patient was very distressed. Muneer et al. described a failed percutaneous puncture attempt due to the needle's inability to reach the balloon [7]. The puncture was performed by a doctor with a lot of puncture experience in this case. The key to a successful puncture appears to be careful positioning with the help of ultrasound.

**Table 1:** Characteristics of cases in which the balloon could not be deflated

Author	Reported year	Gender	R/L	The process of removing the catheter
Muneer et al. [7]	2002	Male	R	A flush of the catheter Percutaneous puncture Endoscopic incision (√)
Viswanatha et al. [8]	2013	Female	R	Cut the balloon channel Slow passive deflation Ureteroscopy forceps (√)
Crawford et al. [6]	2015	Female	R	Cut the balloon channel Burst the balloon by inserting a needle into the balloon channel A flush of the catheter Burst the balloon by inserting a guidewire into the balloon channel (√)
Smekal et al. [2]	2020	Female	R	Cut the balloon channel Burst the balloon by inserting a guidewire into the balloon channel Endoscopic laser puncture (√)
Cho et al. [3]	2021	Male	R	Slow passive deflation (√)

*R* Right ureter; *L* Left ureter

√ = Procedures that lead to successful catheter removal

Patients with a history of bladder surgery, intravesical instillation, neurogenic bladder, and sensory disorder, particularly women, are at a higher risk of having a catheter misplaced into the ureter. Additionally, empty bladder catheterization is thought to be a risk factor [9]. Before inflating the balloon, a quick check is required after the catheter has been inserted. The flow of urine through the catheter can usually indicate whether the catheter is in place. A saline flush is recommended if the catheter is inserted without enough urine output to determine if the catheter is in place and unobstructed [10]. When patients complain of severe abdominal pain on one side, balloon inflation in the ureter should be considered first. When imaging confirms the presence of a catheter in the ureter and the balloon cannot be deflated, non-invasive interventions such as catheter cutting should be attempted first. If these fail, endoscopic surgery or percutaneous puncture should be performed as soon as possible after a full assessment of the patient's overall health and balloon location. Percutaneous puncture has the advantage over endoscopic procedures in terms of convenience, as patients do not need to go to the operating room and can be treated in the ward. What's more, puncture kits are readily available, whereas endoscopes usually require a appointment in advance, making percutaneous puncture suitable for use in emergency situations and when endoscopes are difficult to access. However, the high difficulty of positioning the balloon and the complications associated with puncture, such as bleeding and adjacent organ injury, limit its application in practical scenarios. When it comes to percutaneous puncture, there are a few things to keep in mind: 1. Any contraindications to puncture should be ruled out before the procedure; 2. Under image guidance, precise and careful positioning is required; 3. After the procedure, check the integrity of the removed balloon to ensure that no free debris remains in the body; 4. After puncture, an abdominal pelvic drainage tube should be inserted [10].

This is the first reported case in which a Foley catheter misplaced into the ureter has been removed by percutaneous puncture. This will not be the last case of aberrant catheterization into a ureter, so it should be of concern to all doctors. The passive “twist” of the catheter could be the cause of the balloon's inability to deflate in this case. It is important to know the patient's overall condition and the location of the catheter in the body when it occurs. Interventions should be phased in from non-invasive to invasive.

## Data Availability

Not applicable.

## Abbreviations

CT: Computed tomography
PTC: Percutaneous transhepatic choledochus
RBC: Red blood cell
FFP: Fresh frozen plasma
